# Serotype Distribution and Antimicrobial Susceptibility of *Streptococcus pneumoniae* in Pre- and Post- PCV7/13 Eras, Taiwan, 2002–2018

**DOI:** 10.3389/fmicb.2020.557404

**Published:** 2020-10-22

**Authors:** Chi-Jung Wu, Jui-Fen Lai, I-Wen Huang, Yih-Ru Shiau, Hui-Ying Wang, Tsai-Ling Lauderdale

**Affiliations:** ^1^National Institute of Infectious Diseases and Vaccinology, National Health Research Institutes, Zhunan, Taiwan; ^2^Department of Internal Medicine, National Cheng Kung University Hospital, College of Medicine, National Cheng Kung University, Tainan, Taiwan

**Keywords:** *Streptococcus pneumoniae*, serotype, PCV7, PCV13, non-vaccine serotype

## Abstract

In Taiwan, the 7-valent pneumococcal conjugate vaccine (PCV7) was introduced in 2006 and a PCV13 national childhood catchup program was implemented in 2013. To delineate the trend of serotype distribution and antimicrobial susceptibility following vaccination programs, we investigated a total of 1845 *Streptococcus pneumoniae* isolates collected biennially between 2002 and 2018 over a 3-month period from 25 hospitals. The number of isolates collected over the years decreased significantly in all age groups, from a total of 320 isolates in 2002 (pre-PCV), to 196 in 2010 (post-PCV7/pre-PCV13), to 89 in 2018 (post-PCV13). Overall, PCV7/PCV13 serotypes comprised 66.9%/76.3%, 53.1%/78.1%, and 15.7%/31.5% of isolates in 2002, 2010, and 2018, respectively. The leading serotypes in the pre-PCV era were 23F, 19F, 6B, and 14, while serotype 19A predominated in the post-PCV7/pre-PCV13 era, but non-vaccine serotypes (NVT) 15A (18.0%) and 23A (15.7%) surpassed 19A (10.1%) to become the top two leading serotypes in 2018. All the major serotypes, including the emergent serotypes 15A and 23A, were multidrug-resistant with high rates of non-susceptibility to β-lactam (except serotype 3) and several non-β-lactam agents. PFGE and MLST revealed that while meropenem-susceptible serotype 15A-ST3058 isolates and a serotype 23A-ST338 clone existed in earlier years, rise and spread of meropenem-non-susceptible serotype 15A-ST63 and serotype 23A-ST166 clones occurred in recent years. We conclude that successive implementation of PCVs has led to a marked decrease in pneumococcal isolate burden, but the replacement by meropenem-non-susceptible NVT 15A and 23A highlights the need for continued local surveillance to track pneumococcal evolution in each region to help vaccine polyvalency decisions.

## Introduction

*Streptococcus pneumoniae* (pneumococcus) is a major bacterial pathogen associated with substantial morbidity and mortality in humans (Harboe et al., 2014; Moore et al., 2014). In the 1980s, multidrug-resistant (MDR) *S. pneumoniae* emerged worldwide resulting in therapeutic challenges (Klugman, 1990). To prevent invasive pneumococcal disease (IPD), 23-valent pneumococcal polysaccharide vaccine (PPV23), and 7-, 10-, and 13-valent pneumococcal conjugate vaccines (PCV7, PCV10, and PCV13) were sequentially launched (Wantuch and Avci, 2018). PCV7 includes serotypes 4, 6B, 9V, 14, 18C, 19F, and 23F, PCV10 includes additional serotypes 1, 5, and 7F, and PCV13 includes additional serotypes 3, 6A, and 19A (Wantuch and Avci, 2018). PCV immunization has resulted in significant decline in the incidence of IPD in many countries but its success has partly been compromised by the emergence of non-vaccine serotype IPD, in particular, MDR serotypes 19A post-PCV7 and 15A post-PCV13 (Harboe et al., 2014; Sheppard et al., 2016; Ladhani et al., 2018; Nakano et al., 2018).

To reduce the incidence of IPD in Taiwan, PCV7, PCV10, and PCV13 were introduced sequentially in late 2005, 2010, and 2011 for use in private sectors and have been freely provided by the government to children at high risks for IPD since 2009, 2010, and 2012, respectively (Lu et al., 2019). The reported uptake for at least 1 dose of PCVs in infants in Taiwan increased from 7.4% in 2006, 26.2% in 2008, 42.7% in 2010, to 53.1% in 2012 (Su et al., 2016). A national PCV13 catch-up program for all 2–5 year-old (y.o.) children was launched in March 2013, then extended to 1–5 y.o. children in 2014. Starting in 2015, PCV13 has been included in the routine immunization schedule for 2, 4, and 12 month-olds, and PCV13 uptake has been >80% after 2015 (Lu et al., 2019). PPV23 was provided free to ≥75 y.o. by a non-governmental organization during 2007-2017 and the overall uptake was reported to be 41% in 2008 (Liao et al., 2010), and 20.7% overall between 2009 and 2013 (Chen et al., 2018). Starting in 2017, PPV23 has been provided by the government to ≥65 y.o. but the uptake data on PPV23 in the >65 y.o. are not currently available. National surveillance on IPD conducted in Taiwan during 2006-2017 observed a decreased incidence of IPD caused by PCV7 serotypes with a concurrent increased incidence of IPD caused by serotype 19A, which was subsequently controlled post-PCV13 (Lu et al., 2019). However, there have been limited reports on the trends of antimicrobial resistance in different pneumococcal serotypes post-PCV13.

Because changes in pneumococcal antimicrobial susceptibility and ongoing serotype replacement with emergence of new resistance lineages could occur under antibiotic and vaccine selective pressures (Hiller and Sa-Leao, 2018; Lo et al., 2019; Ouldali et al., 2019; Pinto et al., 2019), the World Health Organization (WHO) recommends that surveillance of pneumococcal infection should be continued for at least 5 years after implementation of PCV. The Taiwan Surveillance of Antimicrobial Resistance (TSAR) is a longitudinal multicenter surveillance program of clinical isolates to monitor the molecular epidemiology of bacterial pathogens and their trends of antimicrobial resistance (Huang et al., 2014; Wu et al., 2017). To evaluate the long-term impact of PCVs, this study investigated the trends of serotype distribution and antimicrobial resistance among pneumococcal clinical isolates from the 2002-2018 TSAR collection with focus on emerging serotypes and MDR clones in the post-PCV13 era to provide updated information for IPD management and future vaccine considerations.

## Materials and Methods

### Isolate Collection and Identification

*Streptococcus pneumoniae* isolates were collected as part of the TSAR program from July to September biennially between 2002 (TSAR III) and 2018 (TSAR XI). The isolates were from the same 25 hospitals located in different regions of Taiwan (Supplementary Figure S1). The collection protocol was similar for all 9 rounds of TSAR and has been described previously for other streptococci (Huang et al., 2014; Wu et al., 2017). For a detailed isolate collection protocol, please see Supplementary Materials. The isolates were recovered from clinical samples taken as part of standard care and the TSAR project was approved by the Research Ethics Committee of National Health Research Institutes (NHRI), Taiwan (EC960205-E, EC1010602-E, EC1030406-E, and EC1050606-E). All isolates were stored at -80^o^C at NHRI for subsequent testing. Prior to antimicrobial susceptibility testing, the identification of *S. pneumoniae* was confirmed based on colony morphology, α-hemolysis, optochin sensitivity, and/or bile solubility tests. The reagents and media were obtained from BBL (Becton Dickinson Microbiology System, Sparks, MD).

### Antimicrobial Susceptibility Testing (AST)

Minimum inhibitory concentrations (MICs) of antimicrobial agents were determined by broth microdilution using Sensititre standard panels (Trek Diagnostics, Thermo Fisher Scientific, East Grinstead, West Sussex, United Kingdom) following manufacturer’s instructions and CLSI guidelines with MIC results interpreted using CLSI breakpoints (CLSI,, 2019). Antimicrobial agents tested include β-lactams (amoxicillin/clavulanate, penicillin, cefuroxime, cefotaxime, ceftriaxone, cefepime, and meropenem) and non-β-lactams (chloramphenicol, clindamycin, erythromycin, levofloxacin, linezolid, tetracycline, trimethoprim/sulfamethoxazole [TMP/SMX], and vancomycin) throughout the study years, except amoxicillin/clavulanate and clindamycin, which were not tested in 2008 and 2002-2006, respectively.

### Serotyping, Pulsed-field Gel Electrophoresis (PFGE), and Multi-locus Sequence Typing (MLST)

The serotype of pneumococcal isolates was determined by the Quellung reaction using commercial omni, pooled group and selected type antisera (Statens Serum Institut, Copenhagen, Denmark) on all isolates. A multiplex PCR serotyping approach has also been used on all isolates since 2010 (Pai et al., 2006). Isolates before 2010 that were omni-serum-positive but pooled group sera-negative were also subjected to PCR serotyping. Non-typeable (uncapsulated) isolates referred to those tested negative by omni-serum and *A* PCR but positive for autolysin (*lytA*) gene by PCR (Verhelst et al., 2003; Satzke et al., 2013; Wyllie et al., 2017). The *wciG* sequence was analyzed on isolates initially typed as 35B following a previously described protocol to differentiate serotype 35D from 35B (Lo et al., 2018).

Genetic relatedness among isolates of serotypes 15A and 23A was investigated first by PFGE following a previously reported procedure (Huang et al., 2014). MLST was then performed on isolates selected from the PFGE dendrograms and the sequence type (ST) was assigned following the public MLST database^1^ (Enright and Spratt, 1998).

### Data Analysis

Antimicrobial susceptibility analysis was made using the WHONET software (O’Brien and Stelling, 2011). PPV23-only serotypes refer to serotypes that are in the PPV23 but not in PCV13, while non-vaccine serotypes (NVTs) refer to serotypes not included in PCV13 and PPV23. The two-tailed Chi-square test was applied to examine the association between serotypes and specimen types by using the Statistical Package for the Social Sciences version 17.0 (SPSS, Chicago, IL, United States). Significance of differences in frequencies and proportions was tested by the χ^2^ test with Yates’ correction. A *p* value < 0.05 was considered statistically significant.

## Results

### Isolates

A total of 1845 isolates were collected from the same 25 participating hospitals between 2002 and 2018. The isolate demographic data are provided in Supplementary Table S1. Overall, around 24.5%, 7.3%, 28.9%, and 36.7% of isolates were from ≤5, 6–17, 18–64, and ≥65 y.o., respectively. The majority of isolates were from inpatients (63.3%). The most common specimens were from respiratory tract (73.7%), followed by abscess/pus (11.6%) and blood (9.9%). The remaining specimens (4.8%) were mostly from ear (1.5%) and eye (1.1%) with a few from CSF (0.4%).

The number of isolates collected decreased steadily over the years, which was observed in all age groups (Supplementary Table S1). A total of 320 isolates were collected in 2002 (pre-PCV), but decreased to 196 in 2010 (post-PCV7/pre-PCV13), and 89 in 2018 (post-PCV13), which corresponded to a reduction of 38.8% and 72.2% in 2010 and 2018, respectively, compared to 2002 (Supplementary Table S1).

### Serotype Distribution

**FIGURE 1:**
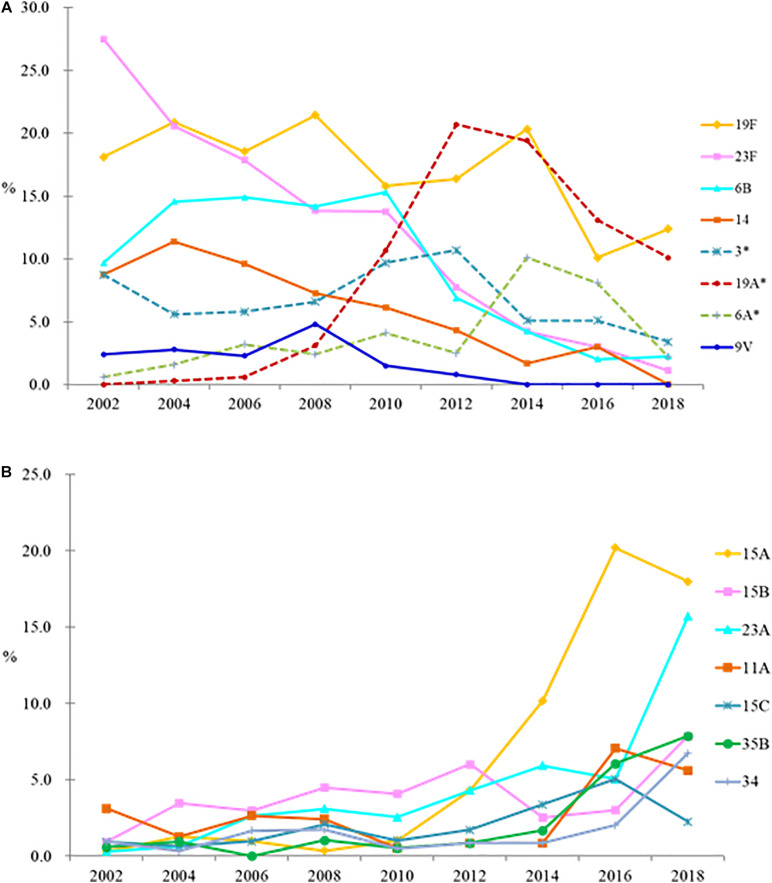
Changes in proportion of PCV7 and PCV13 serotypes **(A)**, and non-PCV13 serotypes **(B)**, among *Streptococcus pneumoniae* isolates in the pre-PCV (2002–2004), PCV7 (2006–2010), and PCV13 (2014–2018) eras. ^∗^, PCV13/non-PCV7 serotypes. Serotypes 4 and 5 plus serogroups 7 and 18 together comprised 1% of all isolates so are not shown, and serotype 1 was not detected.

Changes in the proportion of PCV7 and PCV13 serotypes over the years are shown in Figure 1A, while that of non-PCV13 serotypes are shown in Figure 1B, with detailed serotype distribution data shown in Table 1. In the pre-PCV era (2002-2004), 66.9%–70.3% of the isolates belonged to PCV7 serotypes, among which 23F, 19F, 6B, and 14 predominated. After the introduction of PCV7, the PCV7 serotypes gradually decreased, and PCV13/non-PCV7 serotype 19A emerged in 2008 and became the leading serotype in 2012 (Figure 1A and Table 1). PCV13/non-PCV7 serotype 3 also became the third ranking serotype in 2012. PCV10 provided little additional coverage (<0.1%) compared to PCV7 (data not shown) so is excluded in subsequent discussions. Before PCV13, 73.8%–78.1% of the isolates from 2002-2010 belonged to PCV13 serotypes (Table 1). After the introduction of PCV13, serotypes 19A and 3 started to decline in 2014 while NVT started to increase.

**TABLE 1:** Serotype distribution of 1845 *Streptococcus pneumoniae* isolates from the biennial Taiwan Surveillance of Antimicrobial Resistance (TSAR) program, 2002–2018.

**Serotype**	**No. (%) of isolates**									
	**2002**	**2004**	**2006**	**2008**	**2010**	**2012**	**2014**	**2016**	**2018**	**Total**
	***n* = 320**	***n* = 316**	***n* = 302**	***n* = 289**	***n* = 196**	***n* = 116**	***n* = 118**	***n* = 99**	***n* = 89**	***n* = 1845**
PCV7										
19F	58 (18.1)	66 (20.9)	56 (18.5)	62 (21.4)	31 (15.8)	19 (16.4)	24 (20.3)	10 (10.1)	11 (12.4)	337 (18.3)
23F	88 (27.5)	65 (20.6)	54 (17.9)	40 (13.8)	27 (13.8)	9 (7.8)	5 (4.2)	3 (3.0)	1 (1.1)	292 (15.8)
6B	31 (9.7)	46 (14.6)	45 (14.9)	41 (14.2)	30 (15.3)	8 (6.9)	5 (4.2)	2 (2.0)	2 (2.2)	210 (11.4)
14	28 (8.8)	36 (11.4)	29 (9.6)	21 (7.3)	12 (6.1)	5 (4.3)	2 (1.7)	3 (3.0)	0 (0)	136 (7.4)
9V	8 (2.5)	9 (2.8)	7 (2.3)	14 (4.8)	3 (1.5)	1 (0.9)	0 (0)	0 (0)	0 (0)	42 (2.2)
4	1 (0.3)	0 (0)	2 (0.7)	1 (0.3)	1 (0.5)	0 (0)	0 (0)	0 (0)	0 (0)	5 (0.3)
PCV7 total	214 (66.9)	222 (70.3)	193 (63.9)	179 (61.9)	104 (53.1)	42 (36.2)	36 (30.5)	18 (18.2)	14 (15.7)	1022 (55.4)
PCV13										
3	28 (8.8)	18 (5.7)	18 (6.0)	19 (6.6)	19 (9.7)	13 (11.2)	6 (5.1)	5 (5.1)	3 (3.4)	129 (6.9)
5	0 (0)	0 (0)	0 (0)	0 (0)	1 (0.5)	0 (0)	0 (0)	0 (0)	0 (0)	1 (0.05)
19A	0 (0)	1 (0.3)	2 (0.7)	9 (3.1)	21 (10.7)	24 (20.7)	23 (19.5)	13 (13.1)	9 (10.1)	102 (5.5)
6A	2 (0.6)	5 (1.6)	10 (3.3)	7 (2.4)	8 (4.1)	2 (1.7)	12 (10.1)	8(8.1)^c^	2 (2.2)	56 (3.0)
PCV13 total	244 (76.3)	246 (77.8)	223 (73.8)	214 (74.0)	153 (78.1)	81 (69.8)	77 (65.2)	44 (44.4)	28 (31.5)	1310 (71.0)
PPV23										
15B	3 (0.9)	11 (3.5)	9 (3.0)	13 (4.5)	8 (4.1)	7 (6.0)	3 (2.5)	3 (3.0)	7 (7.9)	64 (3.5)
11A	10 (3.1)	4 (1.3)	8 (2.6)	7 (2.4)	1 (0.5)	1 (0.9)	1 (0.8)	7 (7.1)	5 (5.6)	44 (2.4)
10A	1 (0.3)	4 (1.3)	4 (1.3)	2 (0.7)	0 (0)	1 (0.9)	1 (0.8)	1 (1.0)	0 (0)	14 (0.7)
20	4 (1.3)	3 (0.9)	0 (0)	1 (0.3)	0 (0)	0 (0)	0 (0)	0 (0)	0 (0)	8 (0.4)
22F/A^a^	0 (0)	0 (0)	0 (0)	0 (0)	2 (1.0)	0 (0)	1 (0.8)	0 (0)	0 (0)	3 (0.2)
17F	1 (0.3)	0 (0)	2 (0.7)	2 (0.7)	0 (0)	0 (0)	0 (0)	0 (0)	0 (0)	5 (0.3)
PPV23-only^b^	19 (5.9)	22 (7.0)	23 (7.6)	25 (8.7)	11 (5.6)	9 (7.8)	6 (5.1)	11 (11.1)	12 (13.4)	138 (7.5)
PPV23 total	261 (81.6)	263 (83.2)	236 (78.1)	232 (80.3)	156 (79.6)	88 (75.9)	71 (60.2)	47 (47.5)	38 (42.7)	1392 (75.4)
NVT (non-vaccine type)										
15A	1 (0.3)	4 (1.3)	3 (1.0)	1 (0.3)	2 (1.0)	5 (4.3)	12 (10.2)	20 (20.0)	16 (18.0)	64 (3.5)
23A	1 (0.3)	2 (0.6)	8 (2.6)	9 (3.1)	5 (2.6)	5 (4.3)	7 (5.9)	5 (5.1)	14 (15.7)	56 (3.0)
15C	3 (0.9)	2 (0.6)	3 (1.0)	6 (2.1)	2 (1.0)	2 (1.7)	4 (3.4)	5 (5.1)	2 (2.2)	29 (1.6)
35B	2 (0.6)	3 (0.9)	0 (0)	3 (1.0)	1 (0.5)	1 (0.9)	2 (1.7)	6 (6.1)	7 (7.9)	25 (1.4)
34	3 (0.9)	1 (0.3)	5 (1.7)	5 (1.7)	1 (0.5)	1 (0.9)	1 (0.8)	2 (2.0)	6 (6.7)	25 (1.4)
13	5 (1.6)	1 (0.3)	5 (1.7)	2 (0.7)	2 (1.0)	2 (1.7)	0 (0)	0 (0)	0 (0)	17 (0.9)
35A	4 (1.3)	4 (1.3)	3 (1.0)	2 (0.7)	3 (1.5)	0 (0)	0 (0)	0 (0)	0 (0)	16 (0.9)
22 non-F/A	3 (0.9)	3 (0.9)	3 (1.0)	3 (1.0)	0 (0)	0 (0)	0 (0)	0 (0)	0 (0)	12 (0.7)
Others	15 (4.7)	14 (4.4)	8 (2.6)	4 (1.4)	5 (2.6)	5 (4.3)	3 (2.5)	0 (0)	1 (1.1)	55 (3.0)
NVT total	37 (11.6)	34 (10.8)	38 (12.6)	35 (12.1)	21 (10.7)	21 (18.1)	29 (24.6)	38 (38.4)	46 (51.7)	299 (16.2)
Non-typeable^c^	20 (6.3)	14 (4.4)	18 (6.0)	15 (5.2)	11 (5.6)	5 (4.3)	6 (5.1)	6 (6.1)	3 (3.4)	98 (5.3)

*^a^PPV23 vaccine includes serotype 22F, which is not differentiated from 22A in this study due to its low prevalence. ^b^PPV23-only serotypes refer to 15B, 11A, 10A, 17F, 20, and 22F/A. ^c^Some of the non-typeable isolates might be non-pneumococcal streptococci (Scholz et al., 2012; Sadowy and Hryniewicz, 2020).*

In 2018, NVT serotypes 15A (18.0%) and 23A (15.7%) became the top 2 leading serotypes. Serotype 15A comprised <1.5% of the isolates in each study year between 2002 and 2010, but increased to 4.3% in 2012, 10.2% in 2014, and 20.0% in 2016 (*p* < 0.05), then remained at 18.0% in 2018. Serotype 23A isolates comprised <6% of the 2002–2016 isolates but increased to 15.7% in 2018 (*p* < 0.05) (Figure 1B and Table 1). NVT serotypes 35B (7.9%) and 34 (6.7%) also increased in 2018. All serotype 35B isolates had the wild-type *wciG* sequence thus precluded the possibility of serotype 35D. Overall, only 31.5% of isolates in 2018 were PCV13 serotypes.

**FIGURE 2:**
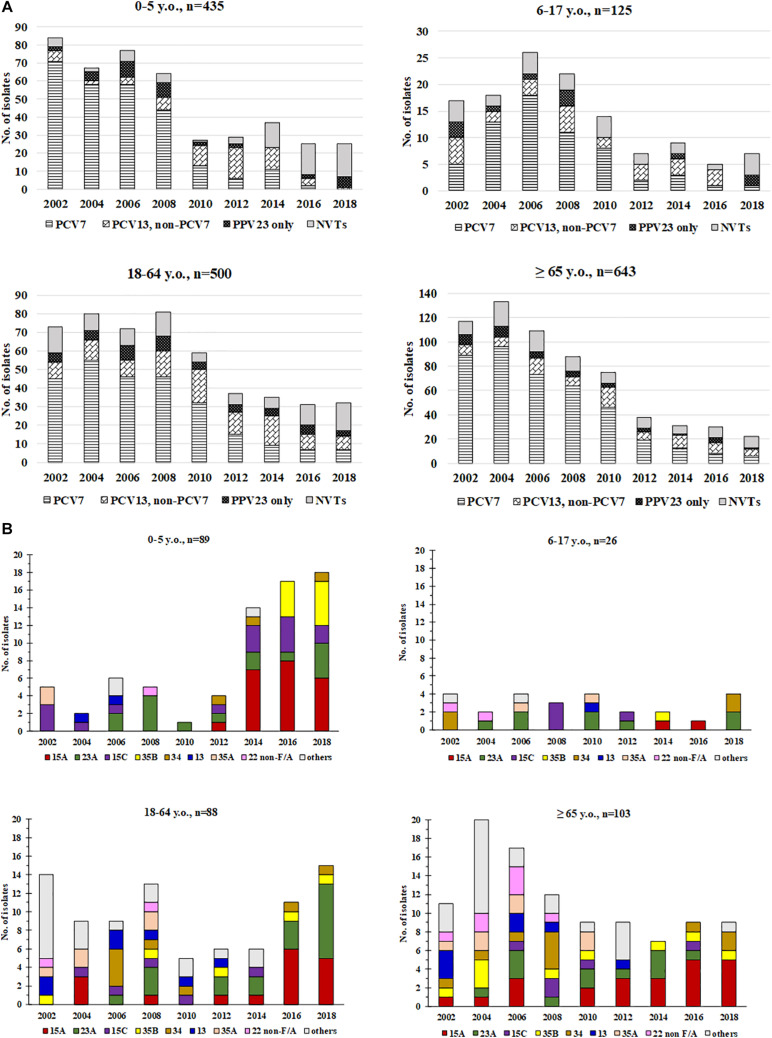
Distribution of **(A)** PCV7, PCV13/non-PCV7, PPV23-only serotypes, and non-vaccine serotypes (NVTs) and **(B)** major non-vaccine serotypes (NVT) among pneumococci from different age groups, 2002-2018. Non-typeable isolates are not included in these figures.

Subgroup analysis showed that concurrent to the decreasing number of isolates, trends of serotype replacement also occurred in all age groups over the years (Figure 2A). However, the change of serotypes was more evident in the <5 y.o. group, among which isolates only one (3.8%) had a PCV13 serotype, compared to 12.5%–54.6% in the other age groups in 2018. Notably, the proportion of PPV23-only serotype isolates in 2018 was lowest in the >65 y.o. group (4.5%) compared to the other age groups (9.4%–25.0%); even when counting just PPV23-only serotypes and NVTs together, the proportion of PPV23-only serotype isolates remained lowest in the >65 y.o. group (10%) compared to the other age groups (16.7%–33.3%). The increase in serotype 15A was observed in all age groups after 2014, while the increase in serotype 23A was observed in the <5, 6–17, and 18–64 y.o. groups (Figure 2B).

Serotype distribution did not differ significantly between the four specimen types, except that (blood vs. non-blood) serotypes 14 (28/182 vs. 108/1663, *p* < 0.001) and 19A (19/182 vs. 83/1663, *p* = 0.002) were more commonly isolated from blood, while serotype 19F was less commonly isolated from blood (21/182 vs. 316/1663, *p* = 0.013). In 2018, 5 (56%) of the 9 blood isolates belonged to non-PCV13 serotypes 11A, 15A, 15B, 23A, and 34. Among the 8 CSF isolates recovered over the years, 5 belonged to PCV13 serotypes (3, 9V, 14, and 19A) before 2010, one serotype 15A isolate from 2004, and two serotype 15B isolates (from 2002 and 2014) (data not shown).

### Antimicrobial Non-susceptibility

The overall non-susceptibility of isolates from each study year is shown in Table 2. All isolates remained susceptible to linezolid and vancomycin. Compared to previous study year, significant changes (*p* < 0.05) in non-susceptibility to different agents were seen in different years (Table 2). For β-lactams, using the non-meningitis breakpoints, despite a decline in 2010, rates of non-susceptibility to penicillin, amoxicillin/clavulanate, cefuroxime (parenteral), cefotaxime, ceftriaxone, cefepime, and meropenem have all increased since 2012, reaching 39.3%, 32.6%, 80.9%, 34.8%, 47.2%, 42.7%, and 70.8% in 2018, respectively. For the non-β-lactam agents, high rates of non-susceptibility to tetracycline (94.2%), erythromycin (93.1%), clindamycin (72.2%), and TMP/SMX (70.3%) were seen, and 37.9% of isolates were chloramphenicol-non-susceptible. Non-susceptibility to clindamycin increased from 2012 (*p* < 0.0.5) on, and while not statistically significant, decreased non-susceptibility to TMP/SMX was observed in 2018.

**TABLE 2:** Antimicrobial non-susceptibility of 1845 *Streptococcus pneumoniae* isolates by collection year, 2002–2018.

**Agent^a^**	**% of non-susceptible isolates**									
	**2002**	**2004**	**2006**	**2008**	**2010**	**2012**	**2014**	**2016**	**2018**	**2002-2018**
	***n* = 320**	***n* = 316**	***n* = 302**	***n* = 289**	***n* = 196**	***n* = 116**	***n* = 118**	***n* = 99**	***n* = 89**	***n* = 1845**
**β-lactam**										
AMC (non-meningitis)	14.0	17.4	9.2^b^	NT^c^	19.4	34.5	39.0	31.3	32.6	20.1
Cefepime (non-meningitis)	28.1	31.0	8.9	21.8	5.1	33.6	45.8	21.2	42.7	23.9
(meningitis)	64.3	64.2	56.6	70.6	24.5	65.5	70.3	57.6	61.8	59.7
Cefotaxime (non-meningitis)	21.8	23.4	8.6	13.5	6.1	37.1	39.8	14.1	34.8	19.3
(meningitis)	63.1	64.5	53.0	63.3	25.0	62.1	68.6	54.6	57.3	57.2
Ceftriaxone (non-meningitis)	24.7	24.1	9.3	23.5	7.1	37.9	49.2	30.3	47.2	23.8
(meningitis)	63.1	65.2	55.3	69.9	32.1	67.2	72.9	57.6	60.7	60.4
Cefuroxime (oral)	66.0	68.1	68.8	74.4	60.2	72.4	82.2	83.8	80.9	70.7
(parenteral)	68.7	70.2	71.2	75.4	67.9	74.1	83.1	83.8	80.9	73.0
Meropenem	61.9	63.3	49.4	66.8	33.7	61.2	73.7	71.7	70.8	59.5
Penicillin (oral)	75.3	78.8	77.8	83.0	76.5	81.0	90.7	90.9	88.8	80.5
(parenteral, non-meningitis)	35.3	41.7	16.6	34.2	16.3	31.9	42.4	40.4	39.3	31.9
(parenteral, meningitis)	75.3	78.8	77.8	83.0	76.5	81.0	90.7	90.9	88.8	80.5
**Non-β-lactam**										
Chloramphenicol	49.1	47.2	41.1	33.6	25.0	31.0	33.9	25.3	24.7	37.9
Clindamycin	NT	NT	NT	66.8	61.7	76.7	83.1	82.8	80.9	72.2
Erythromycin	91.2	92.7	93.0	95.2	90.8	94.0	97.5	93.9	91.0	93.1
Levofloxacin	3.7	5.4	5.6	6.2	9.2	7.8	10.2	5.1	5.6	6.2
Tetracycline	95.0	95.2	95.0	95.9	89.3	90.5	97.5	91.9	93.3	94.2
Trimethoprim/sulfamethoxazole	68.4	71.5	75.2	74.0	67.9	70.7	69.5	66.7	55.1	70.3

*^a^Linezolid and vancomycin were also tested for all study years and no resistance was found. ^b^Underlined, % non-susceptible differed significantly (*p* < 0.05) from previous study year. ^c^NT, Not tested.*

High rates of antimicrobial non-susceptibility were seen in isolates from all age groups (Table 3). However, isolates from the <17 y.o. group overall had higher rates of non-susceptibility to several β-lactam and non-β-lactam agents except levofloxacin (Table 3). Compared to those from the 18–64 and >65 y.o. age groups, isolates from the <17 y.o. had significantly higher rates of non-susceptibility to the following β-lactams: cefepime, cefotaxime, ceftriaxone (all 3 agents by meningitis breakpoints criteria only), cefuroxime (oral criteria), meropenem, and penicillin (oral and meningitis criteria). Rates of non-susceptibility to erythromycin and tetracycline were also significantly higher in the <17 y.o. isolates. Overall, isolates from the 18–64 y.o. had similar rates of non-susceptibility to those of >65 y.o. The only exception is levofloxacin non-susceptibility, which was lowest among isolates in the <17 y.o. group, followed by the 18-64 y.o. group, with a highest rate seen in the >65 y.o. group, at 1.1%, 5.1%, and 11.8%, respectively (*p* < 0.01).

**TABLE 3:** Antimicrobial non-susceptibility of *Streptococcus pneumoniae* from different age groups, 2002–2018.

**Agent^a^**	**% of non-susceptible isolates**			***P***		
	**<17 y.o.**	**18–64 y.o.**	**>65 y.o.**			
	***n* = 586**	***n* = 533**	***n* = 677**	**<17 vs. 18–64**	**18–64 vs. >65**	**<17 vs. >65**
**β-lactam**						
AMC (non-meningitis)	22.0	21.2	16.8	NS	NS	<0.05
Cefepime (non-meningitis)	24.1	23.3	23.9	NS	NS	NS
(meningitis)	66.9	54.3	57.7	<0.01	NS	<0.01
Cefotaxime (non-meningitis)	19.9	19.5	18.6	NS	NS	NS
(meningitis)	65.1	52.0	54.5	<0.01	NS	<0.01
Ceftriaxone (non-meningitis)	24.2	24.0	23.2	NS	NS	NS
(meningitis)	67.4	55.0	58.5	<0.01	NS	NS
Cefuroxime (oral)	76.8	65.8	68.8	<0.01	NS	<0.01
(parenteral)	77.7	68.3	72.6	<0.01	NS	NS
Meropenem	66.7	53.7	57.9	<0.01	NS	<0.01
Penicillin (oral)	87.0	74.3	79.6	<0.01	<0.05	<0.01
(parenteral, non-meningitis)	34.4	30.9	30.2	NS	NS	NS
(parenteral, meningitis)	87.9	77.5	81.7	<0.01	NS	<0.01
**Non-β-lactam**						
Chloramphenicol	37.7	36.8	40.0	NS	NS	NS
Clindamycin	73.9	73.7	71.1	NS	NS	NS
Erythromycin	96.1	91.4	92.0	<0.01	NS	<0.01
Levofloxacin	1.1	5.1	11.8	<0.01	<0.01	<0.01
Tetracycline	94.7	94.2	93.8	NS	NS	NS
TMP/SMX	74.7	65.9	70.0	<0.01	NS	NS

*^a^Amoxicillin/clavulanate (AMC) was not tested in 2008 and clindamycin was not tested in 2002–2006. TMP/SMX, trimethoprim/sulfamethoxazole.*

Antimicrobial non-susceptibility profiles differed between the major serotypes (Table 4). Using the non-meningitis breakpoints, the penicillin non-susceptibility rate was highest in serotype 19A (90.2%), followed by 19F (58.4%), 9V (50.0%), 23F (45.2%), 14 (33.8%), 6A (25.0%), and non-PCV13 serotypes 23A (27.8%), 11A (27.3%), 15B (25.0%), and 15C (24.1%). These serotypes were also associated with higher rates of non-susceptibility to ceftriaxone (19.9%–71.6%) and cefepime (17.2%–67.6%), whereas serotypes 3, 6B, 15A, and 35B had the lowest non-susceptibility (0%–3.8%). The highest rates of meropenem non-susceptibility were found in serotypes 19A (99.0%) and 9V (95.2%), followed by serotypes 6A, 14, 15A, 15B, 15C, 19F, and 23F (55.4%–86.0%). Of note, among serotype 15A isolates from 2002-2010, only one (9.1%, 1/11) was meropenem-non-susceptible but 82.4% (14/15) and 80.6% (29/36) of isolates from 2012-2014 and 2016–2018, respectively, were; and while none of the serotype 23A isolates between 2002-2014 (*n* = 37) were meropenem-non-susceptible, 63.2% (12/19) of those from 2016-2018 were.

**TABLE 4:** Antimicrobial non-susceptibility of major *Streptococcus pneumoniae* serotypes (in order of frequency), 2002–2018.

**Agent^a^**	**% of non-susceptible isolates**														
	**PCV7^b^**					**PCV13**			**Non-PCV13**						
	**19F**	**23F**	**6B**	**14**	**9V**	**3**	**19A**	**6A**	**15A**	**15B**	**23A**	**11A**	**15C**	**35B**	**34**
	***n* = 337**	***n* = 292**	***n* = 210**	***n* = 136**	***n* = 42**	***n* = 129**	***n* = 102**	***n* = 56**	***n* = 64**	***n* = 64**	***n* = 56**	***n* = 44**	***n* = 29**	***n* = 25**	***n* = 25**
β-lactam															
AMC (non-meningitis)	34.2	11.9	10.1	12.1	25.0	1.8	97.8	2.0	14.3	2.0	21.3	8.1	0	0	0
Cefepime (non-meningitis)	37.4	38.3	3.8	21.4	50.0	1.6	67.6	23.2	1.6	25.0	19.6	25.0	17.2	0	4.0
(meningitis)	86.7	87.0	60.5	69.8	90.5	1.6	99.0	55.3	11.0	48.4	21.4	38.6	65.5	36.0	16.0
Cefotaxime (non-meningitis)	31.2	31.2	0.5	14.7	38.1	1.6	62.8	16.1	1.6	23.4	17.9	9.0	17.2	0	4.0
(meningitis)	84.0	83.9	52.4	71.3	88.1	2.4	99.0	57.2	4.7	46.9	21.4	36.4	65.5	32.0	16.0
Ceftriaxone (non-meningitis)	37.7	37.7	1.4	19.9	40.5	1.6	71.6	26.8	3.1	31.3	19.6	22.7	34.5	0	8.0
(meningitis)	88.4	87.7	61.4	73.6	88.1	1.6	99.1	57.2	10.9	56.3	21.4	36.3	65.5	40.0	16.0
Cefuroxime (oral)	94.1	95.6	79.6	80.9	100	1.6	99.0	73.2	82.8	62.5	23.2	38.6	72.4	44.0	28.0
(parenteral)	95.3	96.9	83.3	84.6	100	9.4	99.0	73.2	85.9	62.5	23.2	38.6	72.4	44.0	28.0
Meropenem	86.0	82.2	43.8	69.9	95.2	1.6	99.0	55.4	68.8	56.3	21.4	36.3	69.0	36.0	16.0
Penicillin (oral)	99.7	97.3	92.9	96.3	100	1.6	100	83.9	87.5	85.9	73.2	38.6	96.6	68.0	28.0
(parenteral, non-meningitis)	58.4	45.2	10.5	33.8	50.0	0.8	90.2	25.0	6.2	25.0	17.9	27.3	24.1	0	4.0
(parenteral, meningitis)	99.7	97.3	92.9	96.3	100	1.6	100	83.9	87.5	85.9	91.1	38.7	96.6	68.0	28.0
Non-β-lactam															
Chloramphenicol	19.3	72.6	40.0	35.3	31.0	76.7	1.0	62.5	10.9	64.1	8.9	13.6	72.4	12.0	12.0
Clindamycin	63.1	57.6	47.7	83.7	100	80.0	98.0	66.7	98.2	80.5	93.3	90.9	85.7	50.0	37.5
Erythromycin	99.1	96.9	98.6	95.6	97.6	82.2	100	89.3	95.3	92.2	96.4	97.7	100	84.0	52.0
Levofloxacin	10.1	11.0	2.4	5.1	33.3	2.3	0	1.8	3.1	3.1	0	0	6.9	0	4.0
Tetracycline	97.9	95.2	98.6	91.2	90.5	90.0	96.1	92.9	100	95.3	96.4	97.7	100	68.0	72.0
TMP/SMX	97.1	85.9	96.6	57.4	100	1.6	99.0	71.5	6.2	71.9	23.2	84.1	72.4	44.0	28.0

*^a^Amoxicillin/clavulanate (AMC) was not tested in 2008 and clindamycin was not tested in 2002–2006. TMP/SMX, trimethoprim/sulfamethoxazole. ^b^ PCV7 serotypes are included in PCV13.*

Levofloxacin non-susceptibility was 6.2% overall and was highest in serotype 9V (33.3%) isolates, followed by 23F (11.0%) and 19F (10.1%), but not seen in serotypes 11A, 19A, 23A, and 35B. Two (40.0%) of the five levofloxacin-non-susceptible isolates in 2018 belonged to non-PCV13 serotypes (15A and 15B). Serotype 9V, 11A, 15A, 19A, and 23A isolates had the highest rates of clindamycin non-susceptibility (90.9%–100%), whereas serotypes 3, 15A, and 23A had the lowest rates of TMP/SMX non-susceptibility (1.5%–23.2%).

In the pre-PCV (2002-2004) and post-PCV7/pre-PCV13 (2008-2010) periods, more than 86.0% of penicillin-, ceftriaxone-, cefepime-, or meropenem- non-susceptible isolates belonged to PCV13 serotypes, whereas in the post-PCV13 era (2016-2018) only 62.7%, 56.9%, 57.6%, and 39.6% of penicillin-, cefriaxone-, cefepime-, or meropenem- non-susceptible isolates, respectively, were PCV13 serotypes (Supplementary Table S2). During 2016–2018, in addition to preexisting serotypes 19A, 19F, and 23F, the rise of serotypes 11A, 15B, and 23A contributed substantially to penicillin, ceftriaxone and cefepime non-susceptibility, and these serotypes as well as 15A also contributed to meropenem non-susceptibility.

### Genetic Relatedness of Serotype 15A and 23A Isolates by PFGE and MLST

**FIGURE 3:**
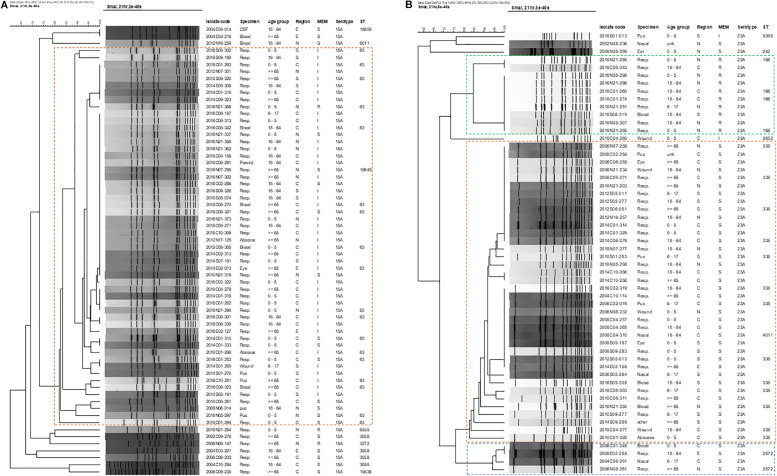
Dendrograms of pneumococcal isolates of emerging meropenem-non-susceptible **(A)** serotype 15A, and **(B)** serotype 23A based on *Sma*I-digested PFGE patterns. Isolate code, the first 4 digits indicate isolation year, followed by region [C (central), N (northern), E (eastern), S (southern)] and 2 digits of hospital number, then 3 digits of isolate number. Other abbreviations: MEM, meropenem; S, susceptible; I: intermediate; R, resistant; ST, sequence type.

The genetic background and clonal relatedness of emergent NVT serotype 15A and 23A isolates were investigated due to their increasing prevalence and meropenem non-susceptibility post-PCV13. Both serotypes 15A and 23A isolates were from different hospitals all over Taiwan, found in every age group, and recovered from various specimen types including blood and CSF (Figures 3A,B).

PFGE revealed that the majority (54/64, 84.3%) of serotype 15A isolates belonged to one main PFGE cluster (isolates sharing >80% similarity), within which multiple isolates had either indistinguishable or >95% similarity in PFGE patterns (Figure 3A). None of the serotype 15A isolates from 2002–2006, but all except one isolate from 2008–2018, belonged to this main cluster, which consist of isolates with ST63 and ST15649 [a single-locus variant (SLV) of ST63], of which most were meropenem-intermediate. In the ten isolates outside the 15A-ST63 main cluster, most were either ST3058 (4 isolates) or SLVs of ST3058 (ST15636 and ST15609), and all were recovered before 2006 and meropenem-susceptible. Two other isolates that were meropenem-resistant were ST6555 and ST3272. Of note, ST15649, ST15636, and ST15609 are new STs that had not been previously reported.

Serotype 23A isolates were more diverse and were grouped into 3 distinct PFGE clusters with most isolates within each cluster being highly clonal (Figure 3B). The largest cluster (*n* = 38) encompasses isolates from different years (2004-2018), all were meropenem-susceptible, and were either ST338 (23A-ST338) or ST4011 (double-locus variant of ST338). Isolates of the second largest cluster (*n* = 10) that were all meropenem-resistant were ST166, and were found only from 2016-2018. Another minor cluster of 4 isolates from earlier years (2002-2008) that were all meropenem-susceptible were ST2572. Two other STs (ST9395 and ST242) were also detected in the remaining 3 isolates of serotype 23A.

## Discussion

Utilizing isolates from a longitudinal multicenter surveillance program (TSAR) covering an extended period from the pre-PCV to the post-PCV13 era (2002–2018), we assessed the impact of PCVs. Our results supported the effectiveness of PCVs, with a downward trend of pneumococcal isolate burden, and an increase of NVTs after sequential introduction of PCV7 and PCV13. However, the effect of PCVs on reducing isolate burden was compromised by increasing β-lactam resistance post-PCV13 owing to the emergence of MDR NVTs.

The decrease in the number of pneumococcal isolates collected could be attributed to a two-step reduction of PCV serotype isolates (PCV7 first, then PCV13), which was in line with reports from around the world (Pilishvili et al., 2010; Kim et al., 2012; Harboe et al., 2014; Moore et al., 2014). We found that after PCV7, 19A and 3 were the most prevalent non-PCV7 serotypes, similar to reports from other Asian countries, Brazil, and Spain (Kim et al., 2012; Guevara et al., 2014; Pinto et al., 2019). Subsequent to PCV13, both serotypes decreased in Taiwan, while studies from Denmark and England found no significant change in serotype 3 IPD incidence (Harboe et al., 2014; Moore et al., 2014; Ladhani et al., 2018). Notably, the protection of PCV13 was greater in ≤5 y.o. children, the primary target of the PCV13 program. The near absence of PCV13 serotypes in this age group in 2018 suggested its direct protective effects. The trend of decreasing isolates and serotype replacement found in the other age groups likely indicated indirect protective effects through herd immunity as observed elsewhere (Harboe et al., 2014; Moore et al., 2014).

While the effects of PCV7 and PCV13 on reducing IPD incidence have been well-documented, the effectiveness of PPV23 has been controversial. Study results against PPV23 included less immunogenicity in children and cost effectiveness economic considerations in older Australians (Chen et al., 2018; Wantuch and Avci, 2018), while large-scale studies supported the protective effects of PPV23 in elderly people against IPD (Shapiro et al., 1991; Ochoa-Gondar et al., 2014). Our observation of a lower proportion of PPV23-only serotype isolates in the >65 y.o. group in 2018 was in agreement with a recent report from Taiwan on a lower IPD risk and related mortality among the elderly who had received PPV23 (Tsai et al., 2015). These results provide support to the recommendation on PPV23 vaccination for the ≥65 y.o^2^.

However, our data from 2016–2018 showed an increase of non-PCV13 serotypes post-PCV13, with serotypes 15A, 23A, 35B, 11A, 15B, and 34 being the most prevalent. A single hospital study in Taiwan in 2012–2014 also found an increased incidence of IPD admissions due to non-PCV13 serotypes, in particular 15A,15B, and 23A (Su et al., 2015). We additionally found the emergence of NVTs 34 and 35B during 2016–2018; both serotypes have also become prevalent in Korea after PCV13 (Park et al., 2019). The occurrence of these non-PCV13 serotypes is worrisome since they were detected in blood isolates in our 2018 collection, indicating their invasiveness. The predominant non-PCV13 serotypes post-PCV13 differed between countries, as reported from England and Wales (8, 12F, 9N, 22F, 15A, 33F, and 23A), the United States (15B/C, 22F, 33F, and 35B/D), Israel (12F, 15B/C, 5, and 33F), South Africa (8, 35B/D, 12F, 15B/C, and 16F), Japan (22F, 15A, and 23A), and Korea (11A, 34, 23A, and 35B) (Moore et al., 2014; Lo et al., 2019; Park et al., 2019; Suzuki et al., 2019). We also did not detect serotype 24F involved in meningitis reported from France and globally distributed invasive serotype 35D post-PCV13 (Lo et al., 2018; Ouldali et al., 2018). Overall, the non-PCV13 serotypes 15A, 23A, 34 and 35B, which increased post-PCV13 observed in our study, were also prevalent in other Asian countries. These findings support the need for local surveillance programs to monitor the dynamics of pneumococcal serotype evolution.

Pneumococci in Taiwan have long been known to have high rates of antimicrobial resistance (Hsueh and Luh, 2002; McDonald et al., 2004). However, data on changes of pneumococcal antimicrobial susceptibility overall over time and on individual serotypes are limited. Based on the higher rates of β-lactam non-susceptibility by meningitis breakpoints criteria (>50%), the combination of a β-lactam and vancomycin for empirical treatment of pneumococcal meningitis is thus reasonable and recommended, especially in the pediatric group among which the β-lactam non-susceptibility was highest. Notably, the observation of higher levofloxacin non-susceptibility in the elderly is in line with our earlier reports revealing higher levofloxacin non-susceptibility among *Haemophilus influenzae* and *Streptococcus agalactiae* isolates from the elderly, which might be the adverse consequences of more frequent use of fluoroquinolone for respiratory illness in this population (Kuo et al., 2014; Wu et al., 2017).

Our study showed that although the overall rates of β-lactam non-susceptibility decreased in 2010 due to the decline of MDR 19F and 23F, the rates have increased since 2012, due in part to the rise of MDR NVTs 15A and 23A, the two most prevalent serotypes in 2018. Most of the serotype 15A isolates belonged to ST63 and had high rates of meropenem non-susceptibility. Post-PCV13 surveillance studies revealed MDR serotype 15A-ST63 isolates to have increased in England in 2011-2014 and clonal spread in Japan in 2012-2014 (Sheppard et al., 2016; Nakano et al., 2018). In Taiwan, while ST338 and ST166 accounted for the majority (74.5%, 35/47) and minority (6.4%, 3/47) of serotype 23A isolates, respectively, during 2012–2014 in one hospital (Su et al., 2015), we observed an increase of a serotype 23A-ST166 MDR meropenem-resistant clone in patients from different hospitals during 2016-2018. ST166 has been associated with serotype 9V isolates in Taiwan, Japan, and Korea before 2014 (Hsieh et al., 2009; Su et al., 2015; Chang et al., 2018; Yun et al., 2018), and in serotype 11A isolates from Korea and Japan more recently (Chang et al., 2018; Kim et al., 2019). However, only one isolate from Japan and two ceftriaxone-non-susceptible isolates from Korea with the serotype 23A-ST166 combination have been described so far but their meropenem susceptibility is unknown (Chang et al., 2018; Choi et al., 2019). Together, these observations suggested emergence of MDR meropenem-non-susceptible serotype 15A-ST63 isolates in different countries, and possible capsular switching of ST166 to serotype 23A isolates followed by local spread of MDR meropenem-resistant serotype 23A-ST166 clone in Taiwan, both of which raise concern since neither serotype is included in the current PCV13.

The introduction of PCVs also influenced the trends of non-β-lactam resistance. The increase of clindamycin non-susceptibility since 2014 and the decreased of SMP-TMX non-susceptibility in 2018 might be attributed to the rise of serotypes 23A and 15A since these serotypes are mostly clindamycin-non-susceptible and TMP/SMX-susceptible. For levofloxacin, the serotypes frequently associated with non-susceptibility i.e., 9V, 19F, and 23F, have gradually decreased in the post-PCV years. Furthermore, levofloxacin non-susceptibility was not found in serotype 19A post-PCV7 and emerging serotypes 11A, 23A, and 35B post-PCV13. Both factors explain stable levofloxacin non-susceptibility rates in 2016-2018 (<6%). Low rates of levofloxacin non-susceptibility were also observed in serotypes 19A and 23A in Korea (Park et al., 2019). However, albeit still at low rate, we have detected levofloxacin-non-susceptible isolates in non-PCV13 serotypes 15A, 15B, 15C, and 34, indicating the need for continued monitoring of levofloxacin resistance.

The selection pressure of pneumococcal vaccine leads to dynamic changes in serotype distribution with subsequent emergence of NVTs (Hiller and Sa-Leao, 2018; Lo et al., 2019; Ouldali et al., 2019). Our surveillance data together with worldwide reports indicated the need of extending vaccine polyvalency. Two higher valency PCV vaccines, PCV15 and PCV20, are currently under development, which includes all PCV13 serotypes plus 22F and 33F in PCV15 and additional serotypes 8, 10A, 11A, 12F, and 15B in PCV20 (Sobanjo-ter Meulen et al., 2015; van der Linden et al., 2019). Although serotypes 33F and 22F were prevalent in western countries, only 3 (0.2%) isolates of 22F/A and 1 isolate of serogroup 33 (these isolates were not further serotyped) were identified here throughout the study years. These two serotypes were also uncommon in South Africa (<0.5%) and Korea (2.4%) post-PCV13 (Dube et al., 2018; Park et al., 2019). The new PCV20 would potentially increase the current PCV13 coverage by 7.7%, 9.0%, 7.7%, and 4.7% in our <5, 6–17, 18–64, and >65 y.o. age groups, respectively. The increased coverage includes 126 PCV20/non-PCV13 isolates comprising 64, 44, 14, 3 and 1 isolates of serotypes 15B, 11A, 10A, and 22F/A, and serogroup 33, respectively, in our collection (no serotypes 8 and 12F were detected in our collection). In terms of invasiveness, multidrug resistance, and wide geographical distribution, serotypes 15A and 23A could be considered for inclusion in the future polyvalent vaccine.

There are certain limitations in this study. First, it is unclear whether some of the non-sterile site isolates were colonizers or true pathogens. Nevertheless, the downward trend of the number of isolates collected was in line with a recent report from a national IPD surveillance program, which revealed a decreased IPD incidence following PCV7 and PCV13 in Taiwan (Lu et al., 2019). Second, the vaccination history of the source patients was not available; hence, it is unknown whether vaccine failure or non-vaccination contributed to the recovery of residual vaccine serotypes following PCVs and PPV23. Third, we cannot exclude the possibility that some of our non-typeable isolates might be non-pneumococci, as recent studies pointed out, and further studies are needed (Scholz et al., 2012; Sadowy and Hryniewicz, 2020).

## Conclusion

In conclusion, successive implementation of PCVs lead to a marked decrease in pneumococcal isolate burden in Taiwan, but the long-term protective effects of PCV13 were compromised by the emergence of NVT isolates, in particular meropenem-non-susceptible serotype15A-ST63 and serotype 23A-ST166 clones that have spread locally. Continuous surveillance is emphasized in order to detect emerging clones and generate knowledge for future expansion of vaccine polyvalency.

## Data Availability Statement

The datasets generated for this study are available from the corresponding author upon reasonable request.

## Author Contributions

JFL performed the majority of the experiments with assistance from IWH, HYW, and YRS. TLL designed and supervised the experiments. CJW and TLL analyzed the data and performed statistical analysis. CJW drafted the manuscript and TLL edited the manuscript. CJW and TLL both revised and finalized the manuscript. All authors approved the final version of the manuscript.

## Conflict of Interest

The authors declare that the research was conducted in the absence of any commercial or financial relationships that could be construed as a potential conflict of interest.

## Footnotes

1 http://pubmlst.org/spneumoniae/

2 https://www.cdc.gov/vaccines/schedules/hcp/imz/adult.html

## Supplementary Material

Supplementary Figure 1 Distribution and locations of hospitals that participated in the Taiwan Surveillance of Antimicrobial Resistance (TSAR) program from 2002 (TSAR III) to 2018 (TSAR XI).

Supplementary Table 1 Source breakdown of 1845 *Streptococcus pneumoniae* isolates from the biennial Taiwan Surveillance of Antimicrobial Resistance (TSAR) program, 2002-2018.

Supplementary Table 2 Distribution of serotypes contributing to β-lactam non-susceptibility (NS) using the non-meningitis criteria among *Streptococcus pneumoniae* isolates in pre-PCV (2002-2004), PCV7 (2008-2010), and PCV13 (2016-2018) eras.
