# Supplementary material for: Serotype Distribution and Antimicrobial Susceptibility of Streptococcus pneumoniae in Pre- and Post- PCV7/13 Eras, Taiwan, 2002–2018
Source: Front Microbiol. 2020 Oct 22;11:557404. doi: 10.3389/fmicb.2020.557404 (PMC7642986; doi:10.3389/fmicb.2020.557404)
Supplement: Supplementary Figure 1 — Distribution and locations of hospitals that participated in the Taiwan Surveillance of Antimicrobial Resistance (TSAR) program from 2002 (TSAR III) to 2018 (TSAR XI). [file Image_1.pdf]

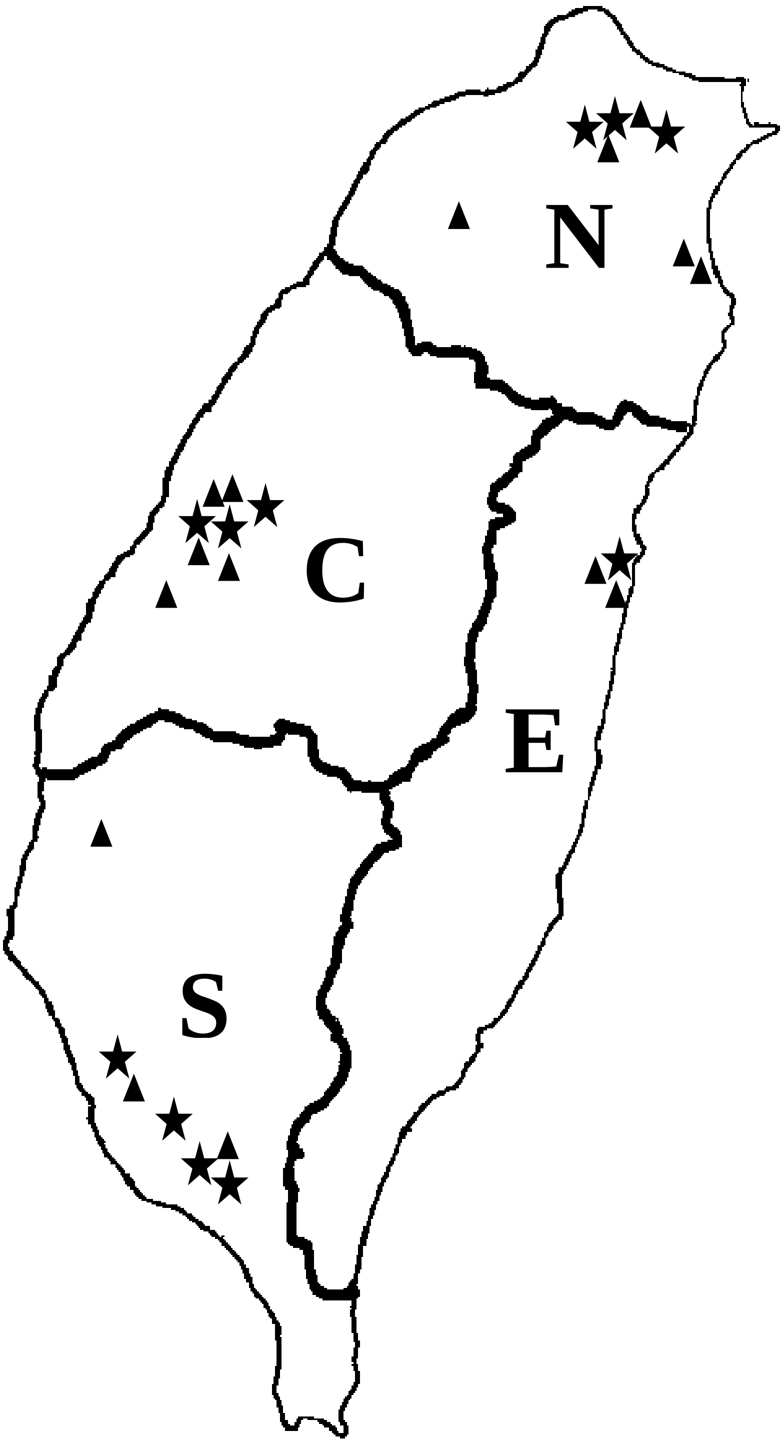
N
C
E
S

Figure S1. Locations of hospitals that participated in the Taiwan Surveillance of Antimicrobial Resistance (TSAR) program from 2002 (TSAR III) to 2018 (TSAR XI). The proximate locations of the hospitals are shown in each region (N, north; C, central; S, South; E, East). Taiwan is a mountainous island and the majority of the people live in the most densely populated western part (north, central and south regions) while the eastern part is the least populated region. Hospital type: star, medical center; triangle, regional hospital.
