# Supplementary material for: Serotype Distribution and Antimicrobial Susceptibility of Streptococcus pneumoniae in Pre- and Post- PCV7/13 Eras, Taiwan, 2002–2018
Source: Front Microbiol. 2020 Oct 22;11:557404. doi: 10.3389/fmicb.2020.557404 (PMC7642986; doi:10.3389/fmicb.2020.557404)
Supplement: Supplementary Table 1 — Source breakdown of 1845 Streptococcus pneumoniae isolates from the biennial Taiwan Surveillance of Antimicrobial Resistance (TSAR) program, 2002-2018. [file Data_Sheet_1.docx]

**Isolate collection**

*Streptococcus pneumoniae* isolates were collected as part of the Taiwan Surveillance of Antimicrobial Resistance (TSAR) program from July and September biennially between 2002 (TSAR III) and 2018 (TSAR XI). The collection protocols were similar for all 9 rounds of TSAR. Briefly, each hospital first collected 200 sequential isolates without specifying bacterial species to include 50 outpatient (including emergency room) isolates, 30 adult ICU and 100 non-ICU inpatient isolates, and 20 pediatric isolates. After completion of the above collection, an additional 20 (for 2002 - 2006) to 50 (2008 - 2018) isolates from blood and sterile body sites were collected, again without specifying bacterial species. To ensure sufficient *S. pneumoniae* isolates were collected, we asked the hospitals to then collect all *S. pneumoniae* isolates for the remainder of the collection months. A total of 1881 isolates were collected from 28 hospitals, 25 of which participated in all 9 rounds of TSAR (except one that did not participate in TSAR V, 2008). The distribution and locations of the 25 hospitals in Taiwan can be seen in Figure S1. For isolate burden and serotype distribution trend analysis, the 1845 isolates from these 25 hospitals were used. Isolates from the other 3 hospitals were excluded from the present study, including one isolate each from two hospitals that participated in only one round of TSAR in different years, and 34 isolates from another hospital which provided isolates between TSAR III (2002) and TSAR VII (2010).

Table S1. Source breakdown of 1845 *Streptococcus pneumoniae* isolates from the biennial Taiwan Surveillance of Antimicrobial Resistance (TSAR) program, 2002-2018.

| Characteristics | No. of isolates (%) | | | | | | | | | |
| --- | --- | --- | --- | --- | --- | --- | --- | --- | --- | --- |
|  | 2002 | 2004 | 2006 | 2008 | 2010 | 2012 | 2014 | 2016 | 2018 | 2002-2018 |
|  | n=320 | n=316 | n=302 | n=289 | n=196 | n=116 | n=118 | n=99 | n=89 | n=1845 |
| Age (y), mean ± SD | 43.4 ± 32.4 | 47.5 ± 31.0 | 43.5 ± 33.0 | 42.9± 31.4 | 51.1 ± 30.1 | 44.7 ± 30.9 | 39.1 ± 32.4 | 42.0 ± 31.1 | 37.5 ± 31.1 | 44.3 ± 31.8 |
| Age group |  |  |  |  |  |  |  |  |  |  |
| ≤ 5 y.o. | 89 (27.8) | 69 (21.8) | 80 (26.5) | 66 (22.8) | 27 (13.8) | 30 (25.9) | 38 (32.2) | 27 (27.3) | 26 (29.2) | 452 (24.5) |
| 6-17 y.o. | 18 (5.6) | 19 (6.0) | 27 (8.9) | 25 (8.7) | 16 (8.2) | 7 (6.0) | 9 (7.6) | 5 (5.1) | 8 (9.0) | 134 (7.3) |
| 18-64 y.o. | 80 (25.0) | 85 (26.9) | 79 (26.2) | 84 (29.1) | 62 (31.6) | 39 (33.6) | 38 (32.2) | 34 (34.3) | 32 (36.0) | 533 (28.9) |
| ≥ 65 y.o. | 124 (38.8) | 136 (43.0) | 116 (38.4) | 94 (32.5) | 81 (41.3) | 40 (34.5) | 33 (28.0) | 31 (31.3) | 22 (24.7) | 677 (36.7) |
| Unknown | 9 (2.8) | 7 (2.2) | 0 (0) | 20 (6.9) | 10 (5.1) | 0 (0) | 0 (0) | 2 (2.0) | 1 (1.1) | 49 (2.7) |
| Patient location |  |  |  |  |  |  |  |  |  |  |
| ER or OPD | 109 (34.1) | 107 (33.9) | 114 (37.7) | 119 (41.2) | 65 (33.2) | 51 (44.0) | 41 (34.7) | 32 (32.3) | 26 (29.2) | 664 (36.0) |
| ICU | 54 (16.9) | 57 (18.0) | 63 (20.9) | 41 (14.2) | 30 (15.3) | 20 (17.2) | 16 (13.6) | 16 (16.2) | 18 (20.2) | 315 (17.1) |
| Inpatient, non-ICU | 157 (49.1) | 148 (46.8) | 125 (41.4) | 129 (44.6) | 101 (51.5) | 44 (37.9) | 55 (46.6) | 50 (50.5) | 43 (48.3) | 852 (46.2) |
| Unknown | 0 (0) | 4 (1.3) | 0 (0) | 0 (0) | 0 (0) | 1 (0.9) | 6 (5.1) | 1 (1.0) | 2 (2.2) | 14 (0.8) |
| Specimen type |  |  |  |  |  |  |  |  |  |  |
| Abscess/Pus | 42 (13.1) | 32 (10.1) | 42 (13.9) | 35 (12.1) | 16 (8.2) | 15 (12.9) | 14 (11.9) | 8 (8.1) | 10 (11.2) | 214 (11.6) |
| Blood | 36 (11.3) | 38 (12.0) | 17 (5.6) | 22 (7.6) | 15 (7.7) | 23 (19.8) | 14 (11.9) | 8 (8.1) | 9 (10.1) | 182 (9.9) |
| CSF | 2 (0.6) | 1 (0.3) | 1 (0.3) | 1 (0.3) | 2 (1.0) | 0 (0) | 1 (0.8) | 0 (0) | 0 (0) | 8 (0.4) |
| Respiratory tract | 220 (68.8) | 237 (75.0) | 231 (76.5) | 218 (75.4) | 153 (78.1) | 70 (60.3) | 83 (70.3) | 80 (80.8) | 67 (75.3) | 1359 (73.7) |
| Others | 20 (6.3) | 8 (2.5) | 11 (3.6) | 13 (4.5) | 10 (5.1) | 8 (6.9) | 6 (5.1) | 3 (3.0) | 3 (3.4) | 82 (4.4) |

Table S2. Distribution of serotypes contributing to nonsusceptibility (NS) to β-lactam antibiotics using the nonmeningitis criteria among *Streptococcus* *pneumoniae* isolates in pre-PCV (2002-2004), post-PCV7/pre-PCV13 (PCV7) (2008-2010), and post-PCV13 (PCV13) (2016-2018) eras.

|  | Penicillin NS, n (%) | | |  | Ceftriaxone NS, n (%) | | |  | Cefepime NS, n (%) | | |  | Meropenem NS, n (%) | | | |
| --- | --- | --- | --- | --- | --- | --- | --- | --- | --- | --- | --- | --- | --- | --- | --- | --- |
| Serotype | Pre-PCV  n=245 | PCV7  n=131 | PCV13  n=75 |  | Pre-PCV  n=155 | PCV7  n=82 | PCV13  n=72 |  | Pre-PCV  n=188 | PCV7  n=73 | PCV13  n=59 |  | Pre-PCV  n=398 | PCV7  n=257 | PCV13  n=134 |  |
| PCV7 |  |  |  |  |  |  |  |  |  |  |  |  |  |  |  |  |
| 19F | 83 (33.9) | 50 (38.2) | 15 (20.0) |  | 37 (23.9) | 34 (41.5) | 13 (18.1) |  | 41 (21.8) | 29 (39.7) | 13 (22.0) |  | 114 (28.6) | 80 (31.1) | 17 (12.7) |  |
| 23F | 92 (37.6) | 20 (15.3) | 4 (5.3) |  | 76 (49.0) | 16 (19.5) | 3 (4.2) |  | 87 (46.3) | 14 (19.2) | 2 (3.4) |  | 142 (35.7) | 40 (15.6) | 4 (3.0) |  |
| 6B | 13 (5.3) | 3 (2.3) | 2 (2.7) |  | 1 (0.6) | 1 (1.2) | 1 (1.4) |  | 5 (2.7) | 1 (1.4) | 0 (0) |  | 34 (8.5) | 32 (12.5) | 2 (1.5) |  |
| 14 | 28 (11.4) | 10 (7.6) | 2 (2.7) |  | 17 (11.0) | 5 (6.1) | 2 (2.8) |  | 23 (12.2) | 3 (4.1) | 0 (0) |  | 52 (13.1) | 20 (7.8) | 2 (1.5) |  |
| 9V | 17 (6.9) | 4 (3.1) | 0 (0) |  | 12 (7.7) | 4 (4.9) | 0 (0) |  | 16 (8.5) | 4 (5.5) | 0 (0) |  | 17 (4.3) | 15 (5.8) | 0 (0) |  |
| PCV13 |  |  |  |  |  |  |  |  |  |  |  |  |  |  |  |  |
| 5 | 0 (0) | 0 (0) | 0 (0) |  | 0 (0) | 0 (0) | 0 (0) |  | 0 (0) | 0 (0) | 0 (0) |  | 0 (0) | 0 (0) | 0 (0) |  |
| 3 | 0 (0) | 0 (0) | 0 (0) |  | 1 (0.6) | 0 (0) | 0 (0) |  | 1 (0.5) | 0 (0) | 0 (0) |  | 1 (0.3) | 0 (0) | 0 (0) |  |
| 19A | 0 (0) | 26 (19.8) | 21 (28.0) |  | 0 (0) | 10 (12.2) | 19 (26.4) |  | 0 (0) | 10 (13.7) | 17 (28.8) |  | 1 (0.3) | 29 (11.3) | 22 (16.4) |  |
| 6A | 2 (0.8) | 4 (3.1) | 3 (4.0) |  | 1 (0.6) | 2 (2.4) | 3 (4.2) |  | 2 (1.1) | 2 (2.7) | 2 (3.4) |  | 3 (0.8) | 5 (1.9) | 6 (4.5) |  |
| Non-PCV13 |  |  |  |  |  |  |  |  |  |  |  |  |  |  |  |  |
| 11A | 0 (0) | 3 (2.3) | 7 (9.3) |  | 0 (0) | 3 (3.7) | 5 (6.9) |  | 1 (0.5) | 3 (4.1) | 5 (8.5) |  | 2 (0.5) | 3 (1.2) | 9 (6.7) |  |
| 15B | 4 (1.6) | 4 (3.1) | 4 (5.3) |  | 4 (2.6) | 3 (3.7) | 7 (9.7) |  | 4 (2.1) | 4 (5.5) | 5 (8.5) |  | 4 (1.0) | 12 (4.7) | 9 (6.7) |  |
| 15A | 0 (0) | 0 (0) | 4 (5.3) |  | 0 (0) | 0 (0) | 2 (2.8) |  | 0 (0) | 0 (0) | 1 (1.7) |  | 0 (0) | 0 (0) | 29 (21.6) |  |
| 23A | 0 (0) | 0 (0) | 10 (13.3) |  | 0 (0) | 0 (0) | 11 (15.3) |  | 0 (0) | 0 (0) | 11 (18.6) |  | 0 (0) | 0 (0) | 12 (9.0) |  |
| 15C | 1 (0.4) | 3 (2.3) | 1 (1.3) |  | 1 (0.6) | 2 (2.4) | 3 (4.2) |  | 1 (0.5) | 1 (1.4) | 1 (1.7) |  | 1 (0.3) | 4 (1.6) | 7 (5.2) |  |
| 34 | 0 (0) | 0 (0) | 1 (1.3) |  | 0 (0) | 0 (0) | 2 (2.8) |  | 0 (0) | 0 (0) | 1 (1.7) |  | 0 (0) | 0 (0) | 3 (2.2) |  |
| 13 | 0 (0) | 2 (1.5) | 0 (0) |  | 0 (0) | 1 (1.2) | 0 (0) |  | 0 (0) | 0 (0) | 0 (0) |  | 0 (0) | 2 (0.8) | 0 (0) |  |
| 35A | 1 (0.4) | 0 (0) | 0 (0) |  | 1 (0.6) | 0 (0) | 0 (0) |  | 1 (0.5) | 0 (0) | 0 (0) |  | 1 (0.3) | 0 (0) | 0 (0) |  |
| 35B | 0 (0) | 0 (0) | 0 (0) |  | 0 (0) | 0 (0) | 0 (0) |  | 0 (0) | 0 (0) | 0 (0) |  | 0 (0) | 0 (0) | 8 (6.0) |  |
| others | 3 (1.2) | 1 (0.8) | 0 (0) |  | 3 (1.9) | 1 (1.2) | 0 (0) |  | 2 (1.1) | 2 (2.7) | 0 (0) |  | 5 (1.3) | 5 (1.9) | 0 (0) |  |
| Nontypeable | 1 (0.4) | 1 (0.8) | 1 (1.3) |  | 1 (0.6) | 0 (0) | 1 (1.4) |  | 4 (2.1) | 0 (0) | 1 (1.7) |  | 21 (5.3) | 10 (3.9) | 4 (3.0) |  |
| PCV7 | 233 (95.1) | 87 (66.4) | 23 (30.7) |  | 142 (91.6) | 60 (73.2) | 19 (26.4) |  | 172 (91.5) | 51 (69.9) | 15 (25.4) |  | 359 (90.2) | 187 (72.8) | 25 (18.7) |  |
| PCV13 | 235 (95.9) | 117 (89.3) | 47 (62.7) |  | 144 (92.9) | 72 (87.8) | 41 (56.9) |  | 175 (93.1) | 63 (86.3) | 34 (57.6) |  | 364 (91.5) | 221 (86.0) | 53 (39.6) |  |
